# Supplementary material for: Diagnostic Accuracy of PET/CT or PET/MRI Using PSMA-Targeting Radiopharmaceuticals in High-Grade Gliomas: A Systematic Review and a Bivariate Meta-Analysis
Source: Diagnostics (Basel). 2022 Jul 8;12(7):1665. doi: 10.3390/diagnostics12071665 (PMC9323081; doi:10.3390/diagnostics12071665)
Supplement: Supplementary file 1 [file diagnostics-12-01665-s001.zip › diagnostics-1799766-supplementary.pdf]

PRISMA-DTA Checklist

Section/topic	#	PRISMA-DTA Checklist Item	Reported on page #
**TITLE / ABSTRACT**			page #1
Title	1	Identify the report as a systematic review (+/- meta-analysis) of diagnostic test accuracy (DTA) studies.	√
Abstract	2	Abstract: See PRISMA-DTA for abstracts.	√
**INTRODUCTION**			page #2
Rationale	3	Describe the rationale for the review in the context of what is already known.	√
Clinical role of index test	D1	State the scientific and clinical background, including the intended use and clinical role of the index test, and if applicable, the rationale for minimally acceptable test accuracy (or minimum difference in accuracy for comparative design).	√
Objectives	4	Provide an explicit statement of question(s) being addressed in terms of participants, index test(s), and target condition(s).	√
**METHODS**			page #2-4
Protocol and registration	5	Indicate if a review protocol exists, if and where it can be accessed (e.g., Web address), and, if available, provide registration information including registration number.	√
Eligibility criteria	6	Specify study characteristics (participants, setting, index test(s), reference standard(s), target condition(s), and study design) and report characteristics (e.g., years considered, language, publication status) used as criteria for eligibility, giving rationale.	√
Information sources	7	Describe all information sources (e.g., databases with dates of coverage, contact with study authors to identify additional studies) in the search and date last searched.	√
Search	8	Present full search strategies for all electronic databases and other sources searched, including any limits used, such that they could be repeated.	√
Study selection	9	State the process for selecting studies (i.e., screening, eligibility, included in systematic review, and, if applicable, included in the meta-analysis).	√
Data collection process	10	Describe method of data extraction from reports (e.g., piloted forms, independently, in duplicate) and any processes for obtaining and confirming data from investigators.	√
Definitions for data extraction	11	Provide definitions used in data extraction and classifications of target condition(s), index test(s), reference standard(s) and other characteristics (e.g. study design, clinical setting).	√
Risk of bias and applicability	12	Describe methods used for assessing risk of bias in individual studies and concerns regarding the applicability to the review question.	√
Diagnostic accuracy measures	13	State the principal diagnostic accuracy measure(s) reported (e.g. sensitivity, specificity) and state the unit of assessment (e.g. per-patient, per-lesion).	√
Synthesis of results	14	Describe methods of handling data, combining results of studies and describing variability between studies. This could include, but is not limited to: a) handling of multiple definitions of target condition. b) handling of multiple thresholds of test positivity, c) handling multiple index test readers, d) handling of indeterminate test results, e) grouping and comparing tests, f) handling of different reference standards	√

PRISMA-DTA Checklist

Section/topic	#	PRISMA-DTA Checklist Item	Reported on page #
Meta-analysis	D2	Report the statistical methods used for meta-analyses, if performed.	√
Additional analyses	16	Describe methods of additional analyses (e.g., sensitivity or subgroup analyses, meta-regression), if done, indicating which were pre-specified.	√
**RESULTS**			page #4-11
Study selection	17	Provide numbers of studies screened, assessed for eligibility, included in the review (and included in meta-analysis, if applicable) with reasons for exclusions at each stage, ideally with a flow diagram.	√
Study characteristics	18	For each included study provide citations and present key characteristics including: a) participant characteristics (presentation, prior testing), b) clinical setting, c) study design, d) target condition definition, e) index test, f) reference standard, g) sample size, h) funding sources	√
Risk of bias and applicability	19	Present evaluation of risk of bias and concerns regarding applicability for each study.	√
Results of individual studies	20	For each analysis in each study (e.g. unique combination of index test, reference standard, and positivity threshold) report 2x2 data (TP, FP, FN, TN) with estimates of diagnostic accuracy and confidence intervals, ideally with a forest or receiver operator characteristic (ROC) plot.	√
Synthesis of results	21	Describe test accuracy, including variability; if meta-analysis was done, include results and confidence intervals.	√
Additional analysis	23	Give results of additional analyses, if done (e.g., sensitivity or subgroup analyses, meta-regression; analysis of index test: failure rates, proportion of inconclusive results, adverse events).	√
**DISCUSSION**			page #11-13
Summary of evidence	24	Summarize the main findings including the strength of evidence.	√
Limitations	25	Discuss limitations from included studies (e.g. risk of bias and concerns regarding applicability) and from the review process (e.g. incomplete retrieval of identified research).	√
Conclusions	26	Provide a general interpretation of the results in the context of other evidence. Discuss implications for future research and clinical practice (e.g. the intended use and clinical role of the index test).	√
**FUNDING**			page #13
Funding	27	For the systematic review, describe the sources of funding and other support and the role of the funders.	√

Adapted From: McInnes MDF, Moher D, Thombs BD, McGrath TA, Bossuyt PM, The PRISMA-DTA Group (2018). Preferred Reporting Items for a Systematic Review and Meta-analysis of Diagnostic Test Accuracy Studies: The PRISMA-DTA Statement. JAMA. 2018 Jan 23;319(4):388-396. doi: 10.1001/jama.2017.19163.

For more information, visit: **www.prisma-statement.org**
